# Serologic Evidence of West Nile Virus Infection in Horses, Yucatan State, Mexico

**DOI:** 10.3201/eid0907.030167

**Published:** 2003-07

**Authors:** María A. Loroño-Pino, Bradley J. Blitvich, José A. Farfán-Ale, Fernando I. Puerto, José M. Blanco, Nicole L. Marlenee, Elsy P. Rosado-Paredes, Julian E. García-Rejón, Duane J. Gubler, Charles H. Calisher, Barry J. Beaty

**Affiliations:** * Colorado State University, Fort Collins, Colorado, USA; † Universidad Autonoma de Yucatan, Merida, Yucatan, Mexico; ‡ Universidad Autonoma de Yucatan, Xmatkuil, Yucatan, Mexico; § Centers for Disease Control and Prevention, Fort Collins, Colorado, USA

**Keywords:** West Nile virus, flavivirus, Mexico, horse, surveillance, dispatch

## Abstract

Serum samples were obtained from 252 horses in the State of Yucatan, Mexico, from July to October 2002. Antibodies to West Nile virus were detected by epitope-blocking enzyme-linked immunosorbent assays in three (1.2%) horses and confirmed by plaque reduction neutralization test. We report the first West Nile virus activity in the State of Yucatan.

West Nile virus (WNV) is a member of the Japanese encephalitis virus complex within the genus *Flavivirus*, family *Flaviviridae* (*1*). The virus is transmitted in natural cycles mainly between mosquitoes and birds, with humans and horses serving as incidental hosts (*2*). WNV was first isolated in 1937 from the blood of a febrile adult human in the West Nile District of Uganda (*3*). This virus has since been reported in Africa, the Middle East, Asia, southern Europe, Australia, and, more recently, North America (*4*,*5*). The initial outbreak of WNV in North America was recognized in New York City in August 1999, with deaths reported in humans, horses, and numerous species of birds. Since then, the geographic distribution of WNV in North America has greatly increased. WNV activity has now been reported in 44 states and the District of Columbia in the United States and in 5 of the 10 Canadian provinces (*6*,*7*).

In response to the incursion and rapid spread of WNV in North America, we established equine and avian infection surveillance in Yucatan State, Mexico, in March 2000. Yucatan State is a likely point of incursion of this virus into Latin America because this area is a principal landfall for many species of birds that migrate from the northeastern and midwestern United States (*8*).

To determine whether WNV had already reached this part of Mexico, we obtained blood samples from 252 domestic horses in 14 study sites from July to October 2002 (Table 1). The age distribution of the horses was 3 months to 25 years, and the mean age was 8.2 years. One hundred and fifty-one horses were male, and 101 were female. All study sites were on privately owned ranches, where the horses were primarily used to perform heavy labor and herd cattle. According to the owners, none of the horses had ever been outside the State of Yucatan. Furthermore, none of the horses had been vaccinated against WNV.

**Table 1:** Study sites and numbers of horses sampled per site, State of Yucatan, Mexico

Study site	Global Positioning System location	No. (%) of horses bled
Acanceh	20° 48' 46" N, 89° 27' 14" W	8 (3.2)
Caucel	21° 00' 53" N, 89° 42' 25" W	1 (0.4)
Hobonil	20° 00' 54" N, 89° 01' 15" W	26 (10.3)
Hunucma	21° 00' 55" N, 89° 52' 28" W	7 (2.8)
Mani	20° 23' 11" N, 89° 23' 37" W	1 (0.4)
Merida	20° 58' 04" N, 89° 37' 18" W	63 (25.0)
Molas	20° 48' 57" N, 89° 37' 55" W	5 (2.0)
Progreso	21° 17' 04" N, 89° 39' 48" W	31 (12.3)
Sierra Papacal	21° 07' 16" N, 89° 43' 41" W	14 (5.6)
Timucuy	20° 48' 34" N, 89° 30' 51" W	5 (2.0)
Tixkokob	21° 00' 08" N, 89° 23' 37" W	15 (6.0)
Tizimin	21° 08' 32" N, 88° 09' 03" W	49 (19.4)
Uman	20° 49' 38" N, 89° 41' 08" W	26 (10.3)
Xbec	21° 14' 54" N, 88° 49' 29" W	1 (0.4)
Total		252 (100)

The climate and topography of the study sites are similar. The climate can be described as tropical. The average annual rainfall in each study site ranges from 600 to 1,100 mm, and the average annual temperature is 26°C. The average elevation is approximately 17 m.

All serum samples were screened for antibodies to flaviviruses by hemagglutination inhibition (HI) assays and epitope-blocking enzyme-linked immunosorbent assays (ELISAs) at the Universidad Autonoma de Yucatan in Merida. HI assays were performed by using Saint Louis encephalitis virus (SLEV) antigen as previously described (*9*). This antigen recognizes cross-reactive HI antibodies to WNV and to other flaviviruses. To preclude nonspecific HI reactions, samples were treated with kaolin, then adsorbed with goose erythrocytes, according to standard methods (*9*). Epitope-blocking ELISAs were performed by using the flavivirus group-reactive monoclonal antibody (MAb), 6B6C-1, or the WNV-specific MAb, 3.1112G as previously described (*10*). The ability of the Mexican horse serum samples to block the binding of MAbs to WNV antigen was compared to the blocking ability of horse serum without antibody to WNV (Vector Laboratories, Burlingame, CA). Data were expressed as relative percentages, and inhibition values >30% were considered to indicate viral antibodies. Recent studies in this laboratory have shown that epitope-blocking ELISA provides a rapid and reliable serologic technique for the detection of WNV antibodies in various vertebrate species, including horses (*10*,*11*).

Six horses had evidence of flavivirus infection by HI assay or ELISA (Table 2). Serum samples from three of these horses (H-117, H-126, and H-252) were positive in the ELISA that used the WNV-specific MAb. H-117 (7-year-old stallion) and H-126 (2-year-old stallion) were both sampled at the Tizimin study site. Neither horse showed signs of illness at the time of serum collection or during the 7 months that followed. Furthermore, neither horse had a history of WNV-like illness. H-252 was a 3-year-old stallion from Caucel that exhibited neurologic and muscular symptoms at the time of sampling; it was euthanized several hours later. We were not able to obtain tissue specimens from this horse postmortem. Of the 252 horses sampled, the only other horse to exhibit signs of clinical illness was H-60, which had signs consistent with gastrointestinal illness.

**Table 2:** Summary of horses with HI assay or epitope-blocking ELISA antibodies to flaviviruses^a^

Horse	Sampling date	Study site	Age (y)	Sex	Clinical symptoms	Outcome	HI assay titer	% inhibition by blocking ELISA^b^	
								3.1112G^c^	6B6C-1^d^
H-60	July 2, 2002	Merida	8	Male	Gastrointestinal (recurrent colic)	Survived	10	0	59
H-117	July 5, 2002	Tizimin	7	Male	None	Survived	10	84	93
H-126	July 5, 2002	Tizimin	2	Male	None	Survived	40	87	93
H-134	July 5, 2002	Tizimin	3	Female	None	Survived	10	11	0
H-141	July 5, 2002	Tizimin	10	Male	None	Survived	80	5	47
H-252	Oct. 15, 2002	Caucel	3	Male	Neurologic and muscular symptoms	Euthanized	20	64	25

^a^HI, hemagglutination-inhibition; ELISA, enzyme-linked immunosorbent assay.

^b^Inhibition values >30% are considered significant.

^c^MAb 3.1112G is WNV-specific.

^d^MAb 6B6C-1 is flavivirus group–reactive.

Serum samples positive for flavivirus antibodies by HI assay or ELISA were tested by plaque reduction neutralization assay (PRNT) to identify the infecting virus. PRNTs were conducted in the BSL-3 facilities at Colorado State University. Serum sample results shown to be negative by HI assay and ELISA were not tested. PRNTs were done by using WNV (strain NY99-35261-11), SLEV (strain TBH-28), Ilhéus virus (ILHV, original strain), and Bussuquara virus (BSQV, strain BeAn-4073). Virus stocks were obtained from the World Health Organization Center for Arbovirus Reference and Research, maintained at the Centers for Disease Control and Prevention, Division of Vector-Borne Infectious Diseases, Fort Collins, CO. We tested serum samples for neutralizing antibodies to SLEV because the virus is enzootic in the Americas and because antibodies to WNV and SLEV often cross-react. Furthermore, horses are susceptible to natural SLEV infections, although clinical manifestations have not been reported (*12*). ILHV and BSQV are also present in the Americas, although neither virus is known to naturally infect horses (*2*). PRNTs were performed by using Vero cells. Serum samples were tested by using a starting dilution of 1:20. Titers were expressed as the reciprocal of serum dilutions yielding >90% reduction in the number of plaques (PRNT_90_).

Neutralizing antibodies to WNV were detected in three horses (Table 3). The PRNT-positive horses were H-117, H-126, and H-252, which exhibited PRNT_90_ antibody titers of 320, >2,560, and 160, respectively. The SLEV, ILHV, and BSQV antibody titers of the three horses were all <20. Therefore, we considered H-117, H-126, and H-252 to be seropositive for WNV because the PRNT_90_ antibody titers for WNV were more than fourfold higher than the other flaviviruses tested. Overall, the PRNT and ELISA data were in concordance; all serum samples that contained neutralizing antibodies to WNV were positive in the assay that used MAb 3.1112G (Tables 2 and 3). However, H-252 was negative in the assay that used MAb 6B6C-1, although the percent inhibition value was close to the diagnostic criterion. The three other horses (H-60, H-134, and H-141) that were positive for flavivirus antibodies by HI assay or ELISA did not have neutralizing antibodies to WNV. H-60 had a low SLEV PRNT_90_ titer, suggesting it had been infected with SLEV or a closely related virus. H-134 exhibited a HI titer of 10 but was negative by the other serologic tests, suggesting that the HI antigen had reacted nonspecifically. H-141 was positive by HI assay and ELISA but had no neutralizing antibodies to any flavivirus tested; thus, the identity of the infecting virus was not determined.

**Table 3:** Neutralizing antibody titers to West Nile, Saint Louis encephalitis, Ilhéus, and Bussuquara viruses in serum samples from six horses^a^

Horse	PRNT_90_ titer			
	WNV	SLEV	ILHV	BSQV
H-60	–^b^	20^c^	–^b^	–
H-117	320	–	–^b^	–
H-126	>2,560	–^b^	–	–
H-134	–	–	–	–
H-141	–	–	–	–
H-252	160	–	–	–

^a^WNV, West Nile virus; SLEV, Saint Louis encephalitis virus; ILHV, Ilhéus virus; BSQV, Bussuquara virus; PRNT, plaque reduction neutralization test; –, <20.

^b^PRNT_80_ titer: 20.

^c^PRNT_80_ titer: 40.

We obtained serologic evidence for antibodies to WNV in Yucatan State, Mexico. The mode of entry of this virus into Yucatan State is not known; however, the virus may have been brought in by birds migrating from the north. We have also detected antibodies to WNV in certain species of migratory birds, which supports this hypothesis. Data from the avian surveillance studies conducted in Yucatan State will be described separately (J.A. Farfán-Ale, unpub. data). We plan to isolate and amplify viral sequences from migratory and resident birds, as well as from specimens from other seropositive animals, to determine the origin of the WNV strain in Yucatan State. We also provide serologic evidence for WNV infection in horses in Coahuila State (*13*). These two reports provide the first published evidence of WNV activity in horses in Mexico. Neutralizing antibodies to WNV have also been detected in a bovine in Chiapas, Mexico, in mid-2001, indicating that the animal had been infected with WNV or a closely related virus (*14*). WNV may become endemic in this country, which demonstrates the importance for continued WNV surveillance in Mexico, and elsewhere in the south.
